# Fusion proteins consisting of Bet v 1 and Phl p 5 form IgE-reactive aggregates with reduced allergenic activity

**DOI:** 10.1038/s41598-019-39798-8

**Published:** 2019-03-08

**Authors:** N. Najafi, G. Hofer, P. Gattinger, D. Smiljkovic, K. Blatt, R. Selb, A. Stoecklinger, W. Keller, P. Valent, V. Niederberger, J. Thalhamer, R. Valenta, S. Flicker

**Affiliations:** 1 Division of Immunopathology, Institute of Pathophysiology and Allergy Research, Center for Pathophysiology, Infectiology and Immunology, Medical University of Vienna, Vienna, Austria; 2 Institute of Molecular Biosciences, BioTechMed Graz, University of Graz, Graz, Austria; 3 Department of Internal Medicine I, Division of Hematology and Hemostaseology, Medical University of Vienna, Vienna, Austria; 4 Department of Otorhinolaryngology, Medical University of Vienna, Vienna, Austria; 5 Department of Molecular Biology, University of Salzburg, Salzburg, Austria; 6 NRC Institute of Immunology FMBA of Russia, Moscow, Russia; 7 Laboratory for Immunopathology, Department of Clinical Immunology and Allergy, Sechenov First Moscow State Medical University, Moscow, Russia

## Abstract

The cross-linking of effector cell-bound IgE antibodies by allergens induces the release of inflammatory mediators which are responsible for the symptoms of allergy. We demonstrate that a recombinant hybrid molecule consisting of the major birch (Bet v 1) and grass (Phl p 5) pollen allergen exhibited reduced allergenic activity as compared to equimolar mixes of the isolated allergens in basophil activation experiments. The reduced allergenic activity of the hybrid was not due to reduced IgE reactivity as demonstrated by IgE binding experiments using sera from allergic patients. Physicochemical characterization of the hybrid by size exclusion chromatography, dynamic light scattering, negative-stain electron microscopy and circular dichroism showed that the hybrid occurred as folded aggregate whereas the isolated allergens were folded monomeric proteins. IgG antibodies raised in rabbits against epitopes of Bet v 1 and Phl p 5 showed reduced reactivity with the hybrid compared to the monomeric allergens. Our results thus demonstrate that aggregation can induce changes in the conformation of allergens and lead to the reduction of allergenic activity. This is a new mechanism for reducing the allergenic activity of allergens which may be important for modifying allergens to exhibit reduced side effects when used for allergen-specific immunotherapy.

## Introduction

The major pollen allergens of birch, Bet v 1, and timothy grass, Phl p 5 were among the first allergens which were characterized by cDNA cloning^1,2^. Bet v 1 and Phl p 5 are clinically important allergens which are recognized by the majority of birch and grass pollen allergic patients^3–5^. Even at very low concentrations they potently induce the cross-linking of effector cell-bound specific IgE antibodies^2,6–8^. Furthermore they induce strong allergic reactions in allergic patients as demonstrated by skin testing and nasal provocation testing^,9,10^.

Bet v 1 and Phl p 5 have therefore been produced as recombinant reference allergens for the standardization of allergen extracts^11^. Assays have been developed to determine Bet v 1 and Phl p 5 concentrations in natural allergen extracts used for diagnostic testing and vaccine production^11^. Moreover, different approaches have been pursued to produce hypoallergenic variants of Bet v 1 and Phl p 5 in order to improve the safety of allergen-specific immunotherapy (AIT)^12–17^. Almost all recombinant Bet v 1 or Phl p 5 hypoallergenic derivatives are characterized by a reduction of the IgE binding capacity compared to the corresponding wild-type allergens^18,19^. These recombinant hypoallergens are thus similar to denatured allergen extracts obtained by chemical treatment (i.e., allergoids) which represent high molecular mass aggregates with reduced IgE reactivity^20^. So far, the only exception to the rule has been a recombinant trimer of Bet v 1 which exhibits an increased IgE reactivity but a reduced allergenic activity when assessed by basophil activation, and skin testing in allergic patients^21^. Accordingly, allergic patients tolerated also high doses of the Bet v 1 trimer in clinical AIT studies^22,23^. An in depth biochemical analysis of the Bet v 1 trimer indicated that the reduction of its allergenic activity was due to the formation of high molecular mass aggregates^24^. It was found that IgE epitopes of these large Bet v 1 aggregates were presented in an orientation that was less effective in cross-linking effector cell-bound IgE than in monomeric Bet v 1^24^. Whether the reduction of allergenic activity through formation of IgE-reactive aggregates is a special feature of the Bet v 1 trimer or represents a mechanism applicable to other allergens has so far remained unanswered. Here we constructed recombinant hybrids consisting of Bet v 1 and Phl p 5. Since each of these allergens occurs as soluble and monomeric protein, we expected the hybrid proteins to remain fully IgE-reactive, allergenic and monomeric as has been observed for hybrids consisting of the grass pollen allergens Phl p 1, Phl p 5, Phl p 2 and Phl p 6^25,26^. However, much to our surprise the Phl p 5-Bet v 1 hybrid formed high molecular aggregates similar to the Bet v 1 trimer, that showed increased IgE reactivity but reduced allergenic activity. The biochemical, biophysical and immunological characterization of the Phl p 5-Bet v 1 hybrid is reported in this study.

## Results

### Expression and purification of Phl p 5-Bet v 1 hybrid molecules

**Figure 1:**
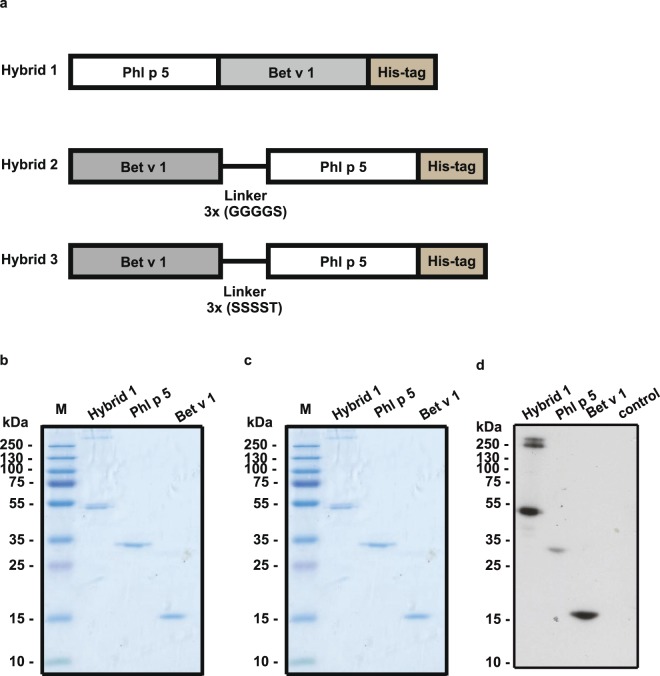
Construction, purification and SDS-PAGE characterization of Phl p 5-Bet v 1 hybrid molecules. (**a**) Schematic representation of recombinant Phl p 5-Bet v 1 hybrid constructs (Hybrids 1–3). Characterization of Hybrid 1 by Coomassie Blue-stained SDS-PAGE under reducing (**b**) and non-reducing conditions (**c**). (**d**) IgE reactivity of nitrocellulose-blotted proteins. Lanes M, molecular mass marker (kDa); lanes Hybrid 1, 1 µg of purified hybrid; lanes Phl p 5, 1 µg of purified rPhl p 5; lanes Bet v 1, 1 µg of purified rBet v 1; lane control, 1 μg rCyp c 1. Full-length gels and blots can be found in the Supplementary Fig. S1A,B.

A recombinant Phl p 5-Bet v 1 hybrid molecule (i.e., hybrid 1) consisting of the complete mature Phl p 5a sequence fused to the Bet v 1a sequence without linker was expressed as C-terminally hexahistidine-tagged protein in BL21 *E. coli* (Fig. 1a). Ni-NTA chromatography yielded approximately 0.5 mg of the recombinant protein per litre of cell culture. A molecular mass of 48 kDa and an isoelectric point of 5.6 were calculated for the recombinant protein. When loaded onto SDS-PAGE, the hybrid showed a band at approximately 48 kDa corresponding to a monomer and high molecular mass (>250 kDa) aggregates were detected under reducing (Fig. 1b) as well as non-reducing conditions (Fig. 1c). Recombinant Phl p 5a and Bet v 1a migrated as distinct bands at approximately 30 kDa and 17 kDa, respectively. Recombinant Phl p 5-Bet v 1 hybrid molecules containing a flexible linker consisting of three copies of GGGGS (i.e., hybrid 2) or a hydrophilic linker consisting of three copies of SSSST (i.e., hybrid 3) were also expressed and purified (Fig. 1a). Hybrid 2 showed a similar pattern with high molecular mass aggregates in SDS-PAGE as hybrid 1 whereas hybrid 3 containing the hydrophilic linker contained less high molecular mass aggregates (data not shown).

Nitrocellulose-blotted hybrid 1 (i.e., the 48 kDa band as well as the high molecular mass aggregates >250 kDa) as well as Phl p 5 and Bet v 1 but not the control allergen (lane control: rCyp c 1) reacted with IgE from a patient with grass pollen and birch pollen allergy (Fig. 1d; Table 1: patient 5).

**Table 1:** Demographic and clinical characterization of patients.

Patient	Age	Sex	Symptoms		Other allergies and symptoms	IgE	
			Birch	Grass		CHIP(ISU)	
						Bet v 1	Phl p 5
1	22	F	RC, asthma	RC, asthma	RC (dog, cat, horse)	38.01	30.86
2	26	M	RC	RC	RC (HDM), OAS (apple)	45.19	54.98
3	32	M	RC, asthma	RC, asthma	RC (cat, horse, HDM, mugwort) OAS (celery, apple, peach)	11.82	23.85
4	32	M	RC	RC	RC (weeds, dog, cat, HDM) OAS (nuts, carrot, celery, apple, melon, soy)	20.62	65.52
5	34	M	RC	RC	RC, asthma (cat), OAS (walnut, hazelnut, pear, apple, peach), Urticaria and dyspnea after consumption of grapes	15.05	2.95
6	24	F	RC, asthma		RC (cat), OAS (hazelnut,walnut, almonds, birch related fresh fruits), AD	3.8	0
7	26	M	RC		RC (animal hair, HDM, moulds)	3.8	0
8	23	F	RC		OAS (apple), RC (HDM, cat)	5.2	0
9	34	M	RC		OAS (apple, hazelnut, figs)	9.1	0
10	52	M	RC		OAS (birch related food)	13.44	0
11	55	F		RC	0	0	4
12	51	F		RC	RC (HDM, cat, ragweed, mugwort)	0	6.06
13	33	M		RC	RC (weeds, HDM)	0	50.5
14	25	M		RC, asthma	OAS (citrus, soy)	0	15.5
15	37	M		RC	0	0	9.7

### The hybrid forms folded high molecular mass aggregates via hydrophobic interactions

**Figure 2:**
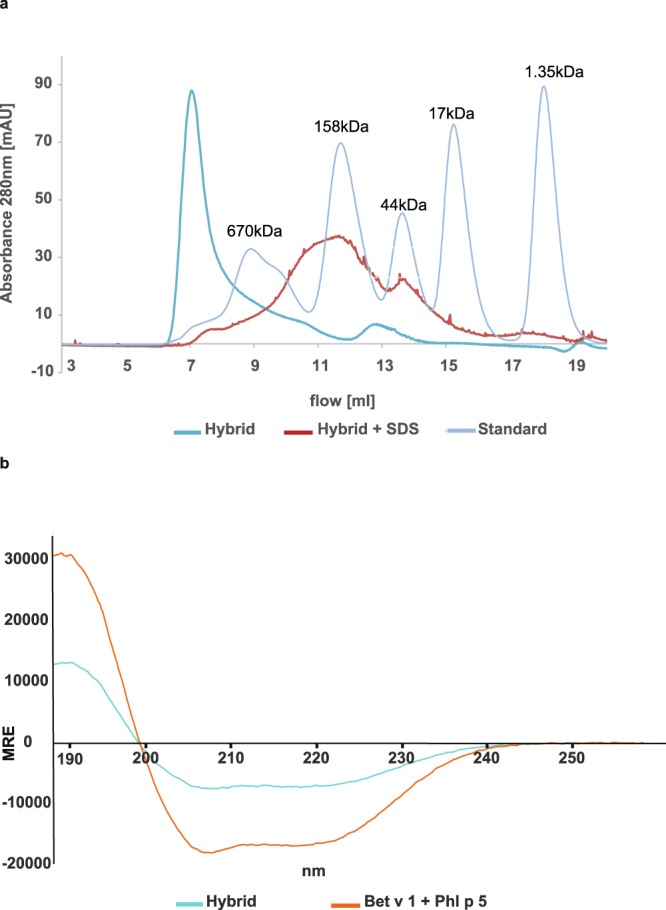
(**a**) Size exclusion chromatography. Elution profile of hybrid 1 run on a gel filtration column in PBS (dark blue) or in PBS containing SDS (red) in comparison to a gel-filtration standard (light blue). X-axis: elution volume in ml, y-axis: absorbance at 280 nm. (**b**) Circular dichroism spectra of the hybrid molecule (blue) in comparison to an equimolar mixture of Bet v 1 and Phl p 5 (red). The mean residue ellipticities (y-axis) are displayed at different wavelengths (x-axis).

Size exclusion experiments (SEC) performed with hybrid 1 demonstrated the presence of two peaks in liquid phase (Fig. 2a). The majority of the sample eluted at the void volume of the column indicating that most of the protein is found in aggregates >1000 kDa. A small peak appearing at an apparent molecular mass of 99 kDa most likely represents the monomer due to the extended hydrodynamic radius previously reported for Phl p 5^27^. To further characterize the larger aggregates a sample was studied using dynamic light scattering and was found to be highly poly-disperse (>25% polydispersity) and the particle size distribution was determined to be between 12 nm and 230 nm, with a mean hydrodynamic radius R_H_ of 47 nm. Neither polydispersity nor size distribution changed considerably upon the change of buffer (*i.e*., 10 mM NaH_2_PO_4_, pH 6 and 1x PBS, pH 7.4) or temperature (*i.e*., 4 °C and 20 °C) (data not shown).

In order to analyze the possible chemical forces responsible for the aggregation of the hybrid molecule, we incubated the protein in 0.15% SDS and performed size exclusion in a buffer containing 0.02% SDS, a strong anionic detergent which dissolved the huge hybrid aggregates (>1000 kDa) into smaller aggregates migrating mainly between 100 kDa and 500 kDa indicating that hydrophobic interactions are responsible for the formation of the aggregates.

The Circular Dichroism (CD) spectrum of hybrid 1 exhibited a similar shape as the mix of allergens but with lower signal intensity (Fig. 2b). CD-based secondary structure prediction of the hybrid 1 indicated a loss of α-helical content compared to the equimolar mix of Phl p 5 and Bet v 1 in favor of an increase in both β-sheet and turn motives. According to calculation by DichroWeb using the CDSSTR method hybrid 1 revealed a 17% α-helical content, 29% β-sheet and 24% turn motives whereas the evaluation for the equimolar mix of the parental molecules indicated 50% α-helical content, 12% β-sheet and 13% turn motives.

### Negative stain electron microscopy shows that the hybrid occurs in the form of large aggregates

**Figure 3:**
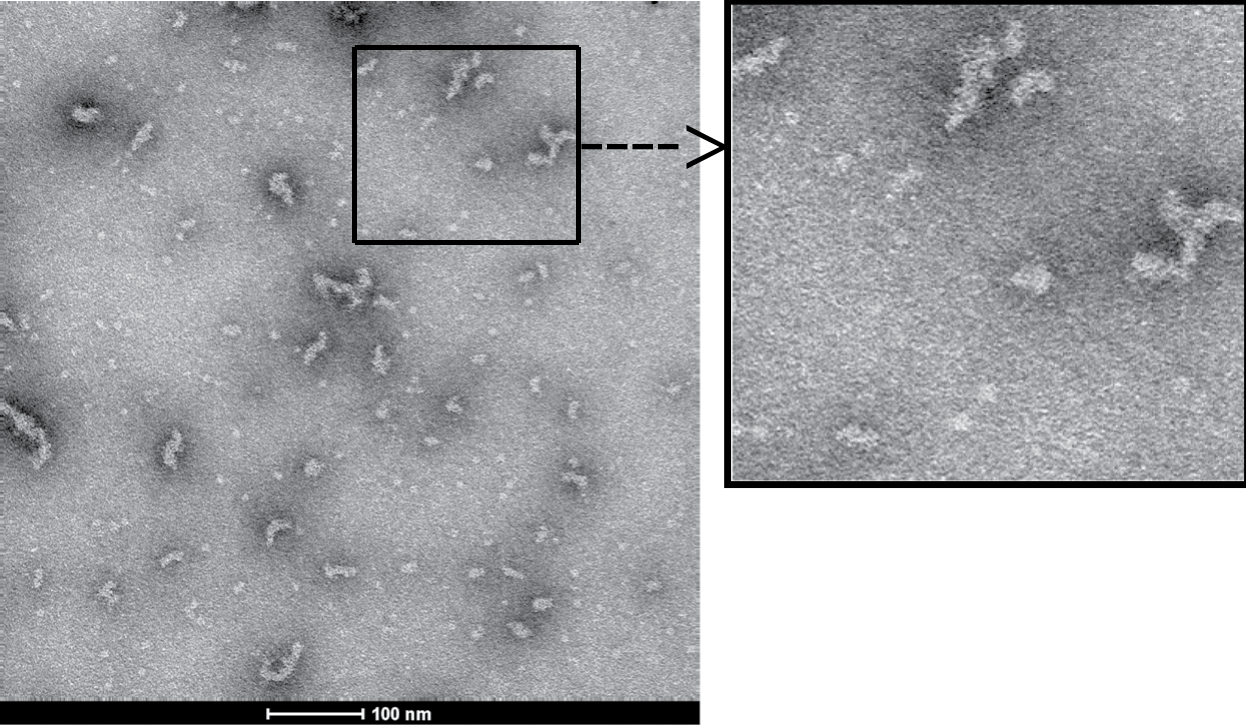
Negative stain electron microscopy of recombinant hybrid 1. Shown is a representative negative stain EM image (left) and a magnification from the image (right). The white bar corresponds to 100 nm.

Negative stain electron microscopy of hybrid 1 revealed the presence of aggregates of different size and eventually monomeric species which corresponds to the results of gel filtration and dynamic light scattering (Fig. 3, left). Monomeric molecules may assume a size of 5–10 nm whereas the large aggregates with a molecular mass of >1000 kDa according to gel filtration analysis may correspond to the 60–70 nm species (Fig. 3, right).

### For birch pollen allergic patients the hybrid exhibits stronger IgE reactivity than an equimolar mix of Phl p 5 and Bet v 1

In order to perform quantitative IgE binding experiments with the hybrid 1 and a mix of Phl p 5 and Bet v 1 we determined conditions of allergen excess at the solid phase. In a pilot experiment we found that 7.5 µM, 15 µM and 30 µM of the proteins on nitrocellulose were sufficient to bind all of the allergen-specific IgE from the sera (data not shown). We then compared the IgE reactivity of hybrid with that of an equimolar mix of Phl p 5 and Bet v 1 with sera from patients who were allergic to Bet v 1 and Phl p 5 (patients 1–5), patients who were only allergic to Bet v 1 (patients 6–10) or only to Phl p 5 (patients 11–15) (Table 2). Interestingly, we found that sera from Bet v 1-allergic patients showed stronger IgE reactivity to the hybrid 1 as compared to the mix of the allergens whereas this was not observed for patients who were only Phl p 5- but not Bet v 1-allergic (Table 2). Increases of IgE reactivity up to 154% (e.g., patient 10) were noted. A similar observation was made earlier for a recombinant Bet v 1 trimer which showed stronger IgE reactivity than the corresponding Bet v 1 allergen^21,24^. Another finding made earlier was that binding of certain mouse and human monoclonal IgG antibodies to Bet v 1 enhanced IgE reactivity of Bet v 1^28–30^, presumably through a change of the conformation of the allergen. We therefore studied if the monoclonal antibody Bip 1^28^ which was shown to enhance patients’ IgE binding to Bet v 1 also has an effect on the IgE recognition of Bet v 1 in the patients in our study (Supplementary Table 1). We found that pre-incubation of Bet v 1 with Bip 1 indeed strongly increased IgE binding to Bet v 1 in 3 out of the 10 birch pollen allergic patients (Supplementary Table 1).

**Table 2:** Quantification of IgE reactivity to dot-blotted hybrid 1 and to an equimolar mixture of Phl p 5 and Bet v 1.

	IgE reactivity (cpm) Phl p 5 + Bet v 1	IgE reactivity (cpm) hybrid	IgE reactivity (cpm) hybrid - (Phl p 5 + Bet v 1)	% difference in IgE reactivity
Patient 1	2937.60	4495.50	1557.90	53%
Patient 2	2402.75	4048.95	1646.20	68%
Patient 3	2010.85	2402.80	391.95	19%
Patient 4	2583.00	5091.00	2508.00	97%
Patient 5	2138.60	2947.05	808.45	37%
**mean**	**2414.56**	**3797.06**	**1382.50**	**55%**
Patient 6	320.80	501.40	180.6	56%
Patient 7	1213.55	1748.70	535.15	44%
Patient 8	68.95	89.85	20.90	30%
Patient 9	261.80	414.25	152.45	58%
Patient 10	152.75	388.85	236.10	154%
**mean**	**403.57**	**628.61**	**225.04**	**68%**
Patient 11	116.80	124.40	7.60	6%
Patient 12	71.10	54.75	−16.35	−23%
Patient 13	3643.90	3392.10	−251.80	−7%
Patient 14	231.80	165.75	−66.05	−29%
Patient 15	843.60	866.05	22.45	2%
**mean**	**981.44**	**920.61**	**−60.83**	**−10%**

Amounts of IgE antibodies (cpm: counts per minute) bound by an equimolar mixture of Phl p 5 and Bet v 1 (column 1) or by hybrid 1 (column 2) are displayed for allergic patients (patients 1–5: birch and grass pollen allergy; patients 6–10: only birch pollen allergy; patients 11–15: only grass pollen allergy). Column 3 shows the differences of IgE reactivity between hybrid and the allergen mix for each patient. Right column: Percentages of higher or lower IgE binding of the hybrid versus the mix.

### Antibodies specific for peptide epitopes show reduced reactivity to the hybrid as compared to the monomeric allergens

**Figure 4:**
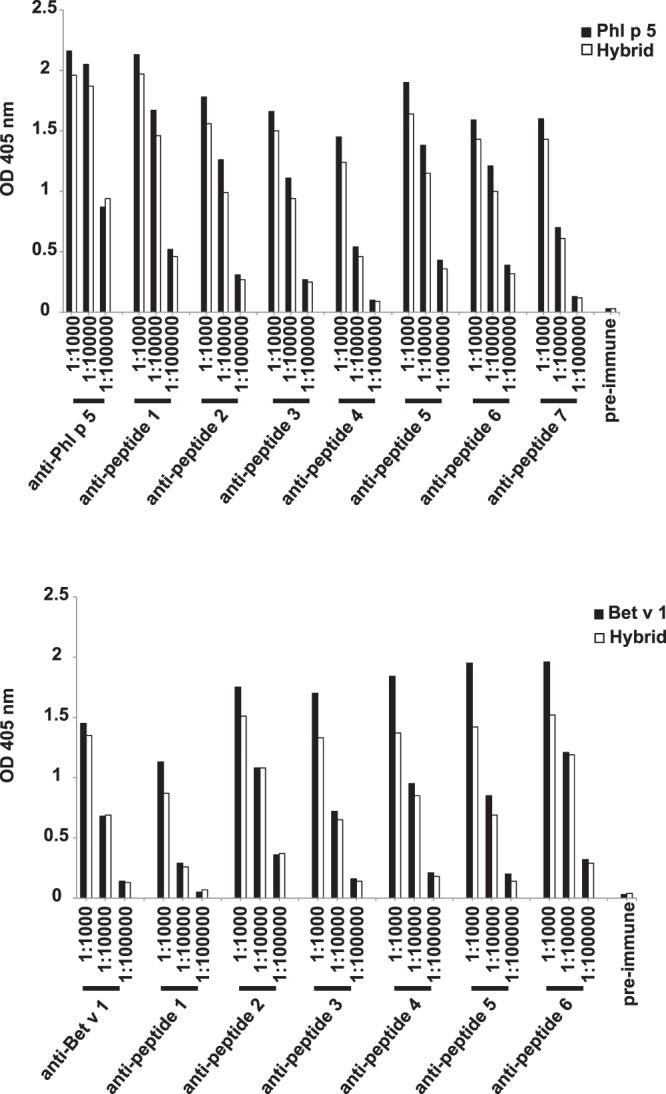
Reactivity of hybrid 1 compared to Phl p 5 (upper panel) and Bet v 1 (lower panel) with different dilutions of rabbit antibodies raised against the complete allergens or against synthetic allergen-derived peptides (x-axes). OD values correspond to the amount of bound antibodies (y–axes).

Next we compared the hybrid 1 with the isolated allergens, Phl p 5 and Bet v 1 regarding reactivity with allergen-specific rabbit antibodies as well as with rabbit antibodies raised against Phl p 5 and Bet v 1-derived peptides (Supplementary Table 2). We found that rabbit IgG antibodies raised against Bet v 1 and Phl p 5 showed a comparable reactivity to the hybrid and to the isolated allergens (Fig. 4). With the exception of the lowest dilution of the Phl p 5-specific antiserum (i.e., dilution 1:1000), the reactivity of rabbit antibodies decreased when the antisera were diluted indicating that the ELISA experiments were done under conditions of antigen excess (Fig. 4). In contrast to allergic patients IgE antibodies which mainly recognize conformational epitopes on Bet v 1 and Phl p 5, the antisera raised against Phl p 5- and Bet v 1- derived peptides are raised exclusively against sequential/ continuous epitopes. Interestingly, the anti-peptide antisera showed a lower reactivity to the hybrid 1 compared to the monomeric allergens which was most pronounced at the dilution of 1:1000 (Fig. 4).

### The hybrid shows reduced allergenic activity compared to an equimolar mix of Phl p 5 and Bet v 1 in birch and grass pollen allergic patients

**Figure 5:**
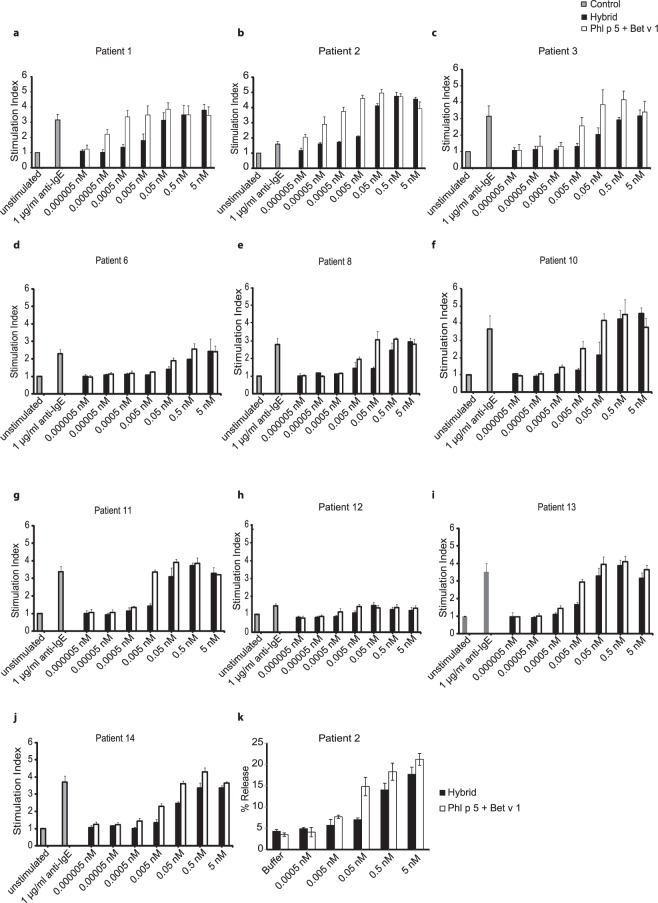
Allergenic activity of hybrid 1 and an equimolar mixture of Phl p 5 and Bet v 1 as determined by CD203c up-regulation on allergic patients basophils. Blood samples from birch and grass pollen allergic (**a–c**), birch pollen allergic (**d**–**f**) or grass pollen allergic patients (**g**–**j**) were exposed to different concentrations of hybrid (black bars) and equimolar mixtures of Phl p 5 and Bet v 1 (open bars), anti-IgE (grey bars) or buffer (unstimulated) (x-axes). The up-regulation of CD203c expression is displayed in the form of stimulation indices (SI) +/− standard deviation (SD) (y-axes) for each of the 10 patients (patients 1, 2, 3, 6, 8, 10, 11, 12, 13, 14) (**a**–**j**). (**k**) RBL cells transfected with human FcεRI were sensitized with serum from a birch and grass pollen allergic patient (1) and then incubated with different concentrations of hybrid or an equimolar mixture of Phl p 5 and Bet v 1 or with buffer alone (x-axis). The release of β-hexosaminidase is shown as percentage of the total β–hexosaminidase contents of the cells +/− SD (y-axis).

To determine the allergenic activity of the hybrid molecule, basophils from birch and/or grass pollen allergic patients were incubated with different concentrations of an equimolar mix of Phl p 5 and Bet v 1 and with the hybrid and the allergen-induced up-regulation of CD203c expression was measured to determine cell activation. We found that the hybrid had an approximately 100-fold lower allergenic activity than the mix of Phl p 5 and Bet v 1 (Fig. 5, Patient 1, 2, 3, 8). An approximately 10-fold lower allergenic activity was observed for patient 6, 10, 11, 13 and 14 (Fig. 5). Thus a reduction of allergenic activity was found for birch (patients 1, 2, 3, 6, 8, 10) as well as for grass pollen allergic patients (patients 1, 2, 3, 11, 13, 14) when comparing the stimulation indices yielded with hybrid versus allergen mix (Fig. 5; Table 1). Basophils from patient 12 were neither activated by the allergens nor by anti-IgE antibodies. Anti-human IgE antibodies used as positive control induced up-regulation of CD203c expression on basophils in all but one donor (i.e., patient 12) and buffer without addition of antigens did not induce up-regulation of CD203c expression (Fig. 5).

**Figure 6:**
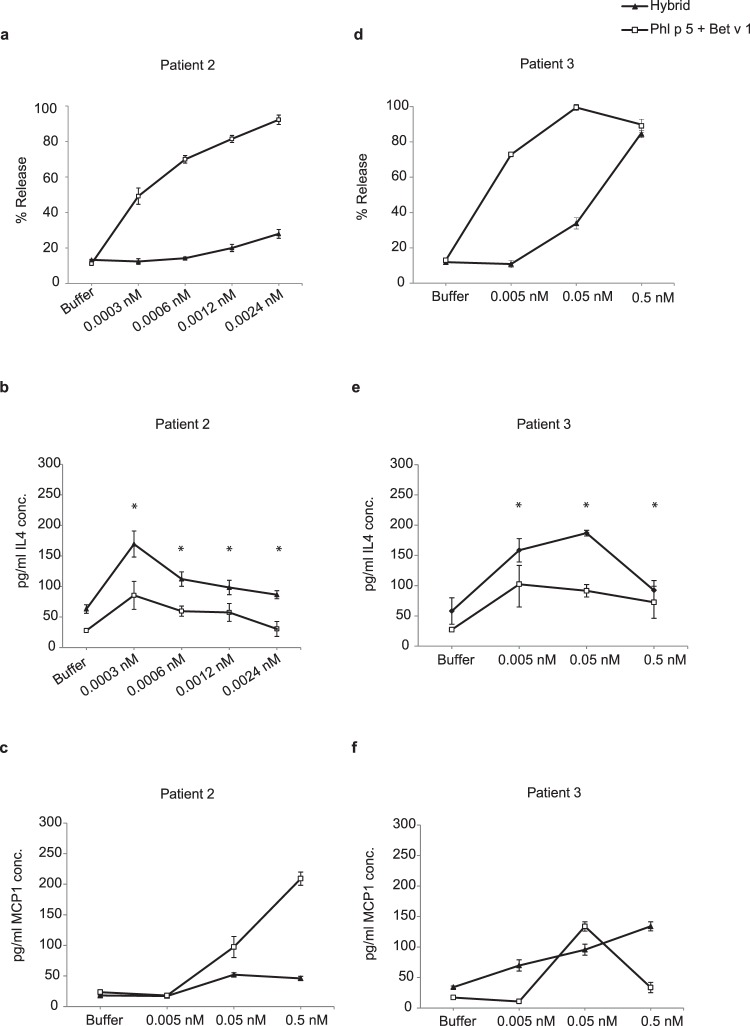
Release of ß-hexosaminidase, IL-4 and MCP-1 from basophils induced by hybrid 1or an equimolar mixture of Phl p 5 and Bet v 1. RBL cells transfected with human FcεRI were sensitized with sera of two birch and grass pollen allergic patients (patient 2: a–c and patient 3:d–f) and then incubated with different concentrations of hybrid or an equimolar mixture of Phl p 5 and Bet v 1 or with buffer alone (x-axis). The release of β-hexosaminidase is shown as percentage of the total β–hexosaminidase contents of the cells+/− SD (y-axis) (**a**,**d**). Release of IL-4 and MCP-1 is shown in (**b**,**e**,**c**,**f**) respectively. *P = 0.05, significant difference.

Similar results as obtained with basophils from allergic patients in full blood under presence of allergen-specific IgG antibodies were observed when rat basophils expressing the human FcεRI were loaded with IgE from a patient allergic to Bet v 1 and Phl p 5 and then washed to remove other antibody isotypes. In the first experiment an approximately 10-fold lower ability to induce the release of ß-hexosaminidase from the cells was found for the hybrid as compared to the allergen mix (Fig. 5k: similar % release obtained at concentrations of 0.05 nM of the allergen mix versus 0.5 nM with the hybrid). For further investigations another RBL cell line i.e., RS-ATL8^31^ was used. We confirmed that the hybrid induces lower ß-hexosaminidase release compared to the mix of Phl p 5 and Bet v 1 in each of the two tested patients (i.e. patient 2, 3) (Fig. 6a,d).

The hybrid induced significantly higher levels of IL- 4 release as compared with the allergen mix, 9 h after allergen challenge. (Fig. 6b,e). However, the allergen mix induced more release of MCP-1, 6 h after allergen challenge as compared to the hybrid (Fig. 6c,f). We found no relevant release of rat TNF-α (data not shown).

## Discussion

Allergen-specific immunotherapy (AIT) is the only disease-modifying treatment for allergy and has long-lasting effects even after discontinuation^32^. The treatment is also more cost-effective than pharmacotherapy^33^ and is based on the modification of the allergic patients’ immune response towards the production of protective IgG antibodies and an alteration of the Th2 immune response^34^. However, the administration of allergens in the course of AIT can induce severe side effects in allergic patients^35^. It is therefore of great interest to develop molecularly defined forms of AIT which are based on recombinant hypoallergenic molecules which exhibit reduced allergenic activity. Several different strategies have been applied to reduce the allergenic activity of allergens, among them fragmentation, the introduction of mutations and the reassembly of allergen in the form of mosaic antigens and peptide-carrier fusion proteins^18,36^. Here we report the expression and biophysical, biochemical and immunological characterization of a recombinant hybrid protein consisting of the major grass pollen allergen, Phl p 5 and the major birch pollen allergen, Bet v 1 which despite increased IgE reactivity was shown to exhibit reduced allergenic activity compared to the mix of the two isolated allergens as demonstrated by basophil activation experiments in patients allergic to Bet v 1 and Phl p 5. The structural and biochemical characterization of the hybrid protein showed that it forms high molecular mass aggregates through tight hydrophobic interactions which remain stable under physiological conditions. To the best of our knowledge, the Phl p 5-Bet v 1 hybrid is the second known example of defined allergens which when expressed as fusion proteins lose their allergenic activity due to formation of aggregates. A similar behavior was found earlier for a recombinant trimer of Bet v 1^21–24^ which had been used successfully for AIT in birch pollen allergic patients in a clinical trial. Surprisingly, the hybrid consisting of Bet v 1 and Phl p 5 described in our study showed increased reactivity with IgE antibodies from birch pollen allergic patients similar as the Bet v 1 trimer but a reduced ability to cross-link IgE antibodies on basophils and thus reduced allergenic activity. Upon IgE cross linking, the hybrid led to a slightly reduced release of MCP-1 compared to the allergen mix; however the hybrid induced significantly more release of IL-4. Nevertheless, the levels of both MCP-1 and IL-4 were very low and thus less important when compared to their different allergenic activity as detected by mediator release.

For the Bet v 1 trimer it has been speculated that the reduced allergenic activity can be due to altered presentation of IgE epitopes in the aggregates^24^. By performing negative-stain electron microscopy we were indeed able to visualize that the hybrid forms stable high molecular mass aggregates providing support for the assumption that reorientation of IgE epitopes explains why the hybrid exhibits reduced allergenic activity. Moreover, results obtained for the hybrid demonstrate that aggregation through stable hydrophobic interaction is not an isolated phenomenon leading to the reduction of allergenic activity of the Bet v 1 allergen only, but also reduces the allergenic activity of another highly allergenic protein, i.e., the Phl p 5 allergen. In fact, Phl p 5 represents one of the most potent allergens which is due to the fact that it is composed of two allergenic domains which are linked by a flexible linker that further increases its allergenic activity^8^. It has therefore been very difficult to reduce the allergenic activity of Phl p 5 by fragmentation or reassembly of fragments^7,37^ or by introducing mutations^17,38^. Our study demonstrates that a considerable reduction of allergenic activity (i.e., >10-fold) can be achieved through molecular aggregation of allergens involving a reorientation of IgE epitopes in an unfavorable position for cross-linking of effector cell-bound IgE antibodies. Furthermore, we found by testing with peptide-specific antisera that certain epitopes may be less accessible in the hybrid than in the isolated allergens.

Our study is in accordance with molecular studies demonstrating that the spatial arrangement of IgE epitopes on allergens have an important impact on the allergenic activity of proteins^39,40^.

Controlled molecular aggregation may thus be used as a new technology for reducing the allergenic activity of allergens and thus for the engineering of a new type of allergy vaccines.

## Materials and Methods

All methods were carried out in accordance with the relevant guidelines and regulations.

### Recombinant expression and purification of hybrid proteins consisting of Phl p 5 and Bet v 1, recombinant allergens

cDNAs coding for hybrid molecules consisting of the major timothy grass pollen allergen Phl p 5a and the major birch pollen allergen Bet v 1a without linker (i.e., hybrid 1), with a flexible linker (3xGGGGS) (i.e., hybrid 2) or with a hydrophilic linker (3xSSSST) (i.e., hybrid 3) between the allergens and a 3′sequence coding for a hexahistidine tag were produced as synthetic genes with codons optimized for expression in *E.coli* and subcloned into the pET17b expression vector (Genscript, New Jersey, USA) (Fig. 1a). The recombinant hybrid molecules were then expressed in *E.coli* BL21 cells and purified by nickel affinity chromatography from the inclusion body fraction according to the manufacturer’s protocol (Quiagen, Hilden, Germany). The purified recombinant hybrid proteins were eluted from the column in 8 M urea, 100 mM NaH_2_PO_4_, 10 mM Tris, pH 4.5, then dialyzed step-wise against 10 mM NaH_2_PO_4_ buffer pH 4.5 in which they remained soluble up to 0.6 mg/ml and could be stored at −20 °C. The purity of the protein preparations was checked by SDS-PAGE and Coomassie Blue staining. The concentrations of the recombinant hybrids were checked by colorimetric detection and quantification of total protein using bicinchoninic acid (BCA) as the detection reagent for Cu1 + (Micro BCA^TM^ Protein Assay Kit, Thermo Scientific, Pierce, Rockford, IL). Recombinant allergens, Bet v 1a and Phl p 5a were obtained from Biomay AG (Vienna, Austria) and shown to represent folded monomeric proteins as previously described^24,27^. For all experiments, the concentrations of the hybrid proteins and the isolated recombinant allergens, rPhl p 5a and rBet v 1a were determined in parallel.

### Size exclusion chromatography

For size exclusion chromatography 500 µl hybrid 1 (0.5 mg/ml 10 mM NaH_2_PO_4_ buffer pH 4.5) were loaded onto a Superdex 200 HR 10/300 column (GE Healthcare, Chalfont St Giles, England) at 4 °C, equilibrated with 1x PBS. The flow rate was 0.4 ml min^−1^. The molecular masses of defined peaks of hybrid 1 were calculated based on the elution profile of a gel filtration standard (670 kDa: Thyroglobulin; 158 kDa: Bovine gamma globulin; 44 kDa: Chicken ovalbumin; 17 kDa: Equine myoglobulin; 1.35 kDa: Vitamin B12) (Biorad, California, USA) performed under identical conditions. In addition, 250 µl hybrid 1 (0.5 mg/ml) that was exposed four days at 4 °C to 0.15% SDS to disrupt possible hydrophobic interactions was loaded onto the same column at 4 °C equilibrated with 1x PBS containing 0.02% w/v SDS. A concentration of 0.15% SDS was used to disrupt possible hydrophobic interactions. As this SDS concentration is below the critical micelle concentration, the formation of micelles and protein-micelle interaction can be ruled out.

### Dynamic light scattering

Dynamic light scattering (DLS) was performed on a DynaPro-99 (Protein Solutions, Lakewood, NJ, USA) using 0.5 mg/ml hybrid 1 protein in a 15 µl cell at either 4 °C or 20 °C in 10 mM NaH_2_PO_4_ pH 6 or 1x PBS. Forty acquisitions 5 s each were carried out with a laser power of 50%. Hydrodynamic radius was calculated using dynamics V6 software (Wyatt Technology Corporation, Santa Barbara, CA).

### Circular dichroism

CD measurements were performed on a Jasco J715 spectropolarimeter (Jasco, Gross-Umstadt, Germany). Spectra were recorded from 190 nm to 260 nm at 20 nm/min and a response time of 2 seconds in a 0.2 mm cell. The hybrid 1 protein was measured at a concentration of 0.5 mg/ml at room temperature. Five individual spectra were baseline corrected and averaged. The results were expressed as the mean residue ellipticity. The secondary structure content of hybrid 1 and the Phl p 5/Bet v 1 mix was calculated using the secondary structure estimation program Dichro Web using the CDSSTR method utilizing reference database 4^41^.

### Negative stain electron microscopy

Negative staining of hybrid 1 was done as described^42^. Briefly, the hybrid molecule was used at a concentration of 10 µg/ml in BSB (borate buffered saline: 10 mM sodium borate, 150 mM NaCl, pH 8.2) and administered to a 2 nm carbon film layer. After washing in 20% BSB and staining with 2% uranyl formate (Polyscience Europe, Eppelheim, Germany), the carbon layer containing the sample was transferred to a T 600H-Cu electron microscope grid (Ted Pella, Redding, CA). Samples were viewed under a trans-emission electron microscope (EM-900 TEM, Carl Zeiss, Oberkochen, Germany).

### Patients and allergen-specific antibodies

Blood samples were obtained from grass and birch pollen allergic patients and non- allergic individuals after informed consent of the patients with the approval of the local ethics committee (Medical University of Vienna, Austria, EK 1641/2014). All experiments were performed in accordance with relevant guidelines and regulations. Grass and birch pollen allergy was diagnosed based on case history, skin testing and the detection of allergen-specific IgE antibodies by allergen microarray (ImmunoCAP ISAC, Thermofisher, Uppsala, Sweden). Table 1 provides a summary of the demographic and clinical characterization of the patients.

Rabbit IgG antibodies specific for Bet v 1 and Phl p 5 had been raised by immunization of rabbits with purified recombinant allergens (rBet v 1, rPhl p 5) or with KLH-coupled allergen-derived peptides using complete and incomplete Freund’s adjuvant (CFA, IFA), respectively (Charles River, Kislegg, Germany) as described^43,44^. Supplementary Tables 2 and 3 provide a summary of the specificities of the peptide-specific antisera^43,44^. The monoclonal mouse antibodies, Bip 1 and 4 A6 were raised against Bet v 1 and Bet v 2, respectively and are described^28,45^.

### Determination of specific IgE reactivity

IgE reactivity of hybrid 1 and of an equimolar mix of Phl p 5 and Bet v 1 was quantified by RAST-based, non-denaturing dot-blot experiments. In pilot experiments the amounts of hybrid and allergens which were dotted onto nitrocellulose were determined to ensure excess of allergen. For this purpose, increasing concentrations (7.5 µM, 15 µM and 30 µM) of hybrid 1 and mix of allergens were dotted and incubated with sera from birch and/or grass pollen allergic patients diluted 1:10 in buffer A (40 mM Na_2_HPO_4_, 0.6 mM NaH_2_PO_4_, 0.5% [v/v] Tween 20, 0.5% [w/v] Bovine serum albumin(BSA), 0.05% [w/v] NaN_3_). Bound IgE antibodies were detected with ^125^I-labeled anti-human IgE (BSM, Diagnostica, Vienna) diluted 1:10 in buffer A and were visualized by autoradiography and quantified (counts per minute: cpm) using a gamma counter (1277 Gammamaster; LKB, Wallac, Gaithersburg, MD, USA).

Based on the results of the pilot experiment 30 µM of the proteins were finally dotted onto nitrocellulose stripes to ensure molar excess of the molecules. Human serum albumin (HSA) served as negative control protein.

### Reactivity of allergen-specific rabbit antibodies with the hybrid and the isolated allergens

ELISA plates (Nunc Maxi-Sorp) were coated with 75 nM hybrid 1, as well as with 75 nM Phl p 5 and 75 nM Bet v 1 (in 100 mM NaHCO_3_, pH = 9.6). Plates were incubated for 3 h at 37 °C, washed twice with PBST, saturated with PBST containing 1% w/v BSA for 3 h at 37 °C and incubated with an anti-Phl p 5-, an anti-Bet v 1-, seven Phl p 5 peptide-specific or six Bet v 1 peptide-specific rabbit antisera diluted 1:1000, 1:10000 and 1:100000. Plates were incubated overnight at 4 °C and washed five times with PBST. Bound antibodies were detected with HRP-labeled anti-rabbit IgG diluted 1:2000 in PBST/0.5% w/v BSA incubated for 2 h at 37 °C. Optical density (OD) measurements were carried out at 405 nm on ELISA reader Spectramax Plus (Molecular Devices). All determinations were performed in duplicates, and results are displayed as mean values with less than 3% variation.

### Measurement of up-regulation of CD203c expression on patients’ basophils by flow cytometry

Peripheral blood samples were obtained from birch and/or grass pollen allergic patients who had been tested for Bet v 1 and Phl p 5-specific IgE reactivity (Table 1). Blood was collected in heparinized tubes. Blood aliquots (90 µl) were incubated with (10 µl) serial dilutions of recombinant hybrid 1 (end concentration: 0.005 pM to 5 nM), equimolar mixtures of Phl p 5 and Bet v 1 (end concentration: 0.005 pM to 5 nM), with anti-IgEmAbE-124.2.8 to the end concentration of 1 µg/ml (Immunotech, Vaudreuil-Dorion, Quebec, Canada) or PBS for 15 min at 37 ^°^C. Then CD203c expression was measured by multicolor flow cytometry as described^46^. In these experiments, mean fluorescence intensities of stimulated (MFIstim) and unstimulated (MFIcontrol) cells were determined by flow cytometry and the up-regulation of CD203c expression was expressed as stimulation index (MFIstim: MFIcontrol) (SI)^46^.

### Rat basophil leukemia cell mediator-release assay

Rat basophil leukemia cells (RBL-2H3) transfected with the human high affinity IgE receptor FcεRI (clone RBL-703/21)^47^, were loaded with serum from a birch and grass pollen allergic patient diluted 1:10 and incubated overnight. Cells were then washed 3 times with Tyrode’s buffer (Sigma-Aldrich, St. Louis, Missouri, USA) and then exposed to different concentrations of either hybrid 1 (0.5 pM to 5 nM) or the equimolar mixture of Phl p 5 and Bet v 1 (0.5 pM to 5 nM)^47^. To increase and stabilize the secretion of ß-hexosaminidase, 50% D2O was added to the Tyrode’s buffer used for the dilution of the hybrid and the mix^48^. For control purposes, IgE-loaded cells were incubated with Tyrode’s buffer only to measure spontaneous release. Total β-hexosaminidase content was determined after lysis of the cells by the addition of Triton-X100 (Sigma-Aldrich) at a final concentration of 1% onto control wells. Allergen-specific release is given as percentage of total mediator content after correction for spontaneous release^47^. In experiments comparing mediators, cytokine and protease release rat basophil leukaemia mast cells (RS-ATL8)^31^ transfected with the human high affinity IgE receptor FcεRI (alpha, beta and gamma), were loaded with serum from birch and grass pollen allergic patients diluted 1:10 and incubated overnight. The release of β-hexosaminidase was measured as described^47^.

TNF-𝛼, IL-4 or MCP-1 releases were determined after different time points (i.e., 1 h, 3 h, 4 h, 6 h, 9 h and 20 h) after allergen challenge^49–51^. Cell culture samples were centrifuged and 50 µl aliquots of each sample were stored at −80 °C until analysis. TNF-𝛼, IL-4 or MCP-1 concentrations were measured using quantitative ELISA assays (R&D SYSTEMS, a biotechne brand, quantkine ELISA, rat TNF-α Immunoassay; Biogems, rat IL-4 pre-coated ELISA Kit and LSBio, Rat CMA1/Mast Cell Chymase ELISA Kit) according to the manufacturer’s instructions. Statistically significant differences of IL-4 release after challenge with hybrid versus allergen mix were determined by paired t-test using Prism 5.04 (GraphPad Software, La Jolla, CA, USA).

## Supplementary information

Supplementary Dataset 1
