# Supplementary material for: Fusion proteins consisting of Bet v 1 and Phl p 5 form IgE-reactive aggregates with reduced allergenic activity
Source: Sci Rep. 2019 Mar 8;9:4006. doi: 10.1038/s41598-019-39798-8 (PMC6408504; doi:10.1038/s41598-019-39798-8)
Supplement: Supplementary file 1 — Supplementary Dataset 1 [file 41598_2019_39798_MOESM1_ESM.pdf]

Fusion proteins consisting of Bet v 1 and Phl p 5 form IgE-reactive aggregates with reduced allergenic activity

N. Najafi[1], G. Hofer[2], P. Gattinger[1], D. Smiljkovic[3], K. Blatt[3], R. Selb[4], A. Stoecklinger[5], W. Keller[2], P. Valent[3], V. Niederberger[4], J. Thalhamer[5], R. Valenta[1, 6], S. Flicker[1*]

[1]Division of Immunopathology, Institute of Pathophysiology and Allergy Research, Center for Pathophysiology, Infectiology and Immunology, Medical University of Vienna, Vienna, Austria
[2]Institute of Molecular Biosciences, BioTechMed Graz, University of Graz, Graz, Austria
[3]Department of Internal Medicine I, Division of Hematology and Hemostaseology, Medical University of Vienna, Vienna, Austria
[4]Department of Otorhinolaryngology, Medical University of Vienna, Vienna, Austria
[5]Department of Molecular Biology, University of Salzburg, Salzburg, Austria
[6]NRC Institute of Immunology FMBA of Russia, Moscow, Russia.

Short title: IgE-reactive aggregates with reduced allergenic activity

Correspondence to:

Sabine Flicker, PhD

Division of Immunopathology

Department of Pathophysiology and Allergy Research

Center for Pathophysiology, Infectiology and Immunology

Medical University of Vienna,

Währinger Gürtel 18-20, A-1090 Vienna, Austria

Tel.: +43-1-40400-51150

Fax: +43-1-40400-51300

e-mail: sabine.flicker@meduniwien.ac.at

Materials and Methods

All methods were carried out in accordance with the relevant guidelines and regulations.

Measurement of Bip 1 binding on serum IgE reactivity to Bet v 1

The effect of Bip 1, a monoclonal Bet v 1-specific mouse IgG on the binding of allergic patients' IgE to Bet v 1 was investigated as described [1]. Aliquots of ten nanogram of rBet v 1 were dotted onto nitrocellulose. Nitrocellulose strips were pre-incubated with Bip 1 hybridoma supernatants or 4A6 cell culture supernatant (negative control) 1:1 diluted in buffer A overnight at 4°C [1, 2]. Nitrocellulose strips were then washed twice for 5 min and once for 30 min in buffer A, and incubated with sera from Bet v 1 allergic patients and control sera without Bet v 1-specific IgE that had been diluted 1: 10 in buffer A. Bound IgE was detected with 125 I-labeled anti-human IgE (BSM) diluted 1:10 in buffer A and were visualized by autoradiography and quantified (counts per minute: cpm) using a gamma counter (1277 Gammamaster; LKB, Wallac).

References

1. Laffer, S., Vangelista, L., Steinberger, P., Kraft, D., Pastore, A., Valenta, R. Molecular characterization of Bip 1, a monoclonal antibody that modulates IgE binding to birch pollen allergen, Bet v 1. J Immunol 157, 4953-4962 (1996).

2. Wiedemann, P., Giehl, K., Almo, S.C., Federov, A.A., Girvin, M. et al. Molecular and structural analysis of a continous birch profilin epitope definded by a monoclonal antibody. J Biol Chem 271, 29915-29921 (1996).

Supplementary Figures and Tables

Supplementary Figure S1. Characterization of hybrid 1 by (**A**) Coomassie Blue-stained SDS-PAGE under reducing (left side) and non-reducing (right side) conditions. (**B**) IgE reactivity of nitrocellulose-blotted proteins (left blot) and negative control (right blot). Lanes M, molecular mass marker (kDa); lanes Hybrid 1, 1µg of purified hybrid; lanes Phl p 5, 1µg of purified rPhl p 5; lanes Bet v 1, 1µg of purified rBet v 1; lane control, 1 µg rCyp c 1; negative control, blot incubated without patient IgE and with ~~125I-labeled~~ anti-IgE antibodies only**.**

Supplementary Table 1. Modulation of patients IgE binding to Bet v 1 by monoclonal antibody Bip 1. Quantification of IgE reactivity (cpm: counts per minute) to Bet v 1 after pre-incubation of Bet v 1 with the monoclonal antibody Bip 1 or after incubation with a control antibody, 4A6 . The middle column displays the calculated cpm difference of the

IgE reactivity to Bet v 1. The percentage increase/decrease of IgE reactivity through Bip 1 is indicated in the right column.

Supplementary Table 2 and 3. Specificities of rabbit antisera raised against Bet v 1- (Table 2) and Phl p 5- (Table 3) derived peptides.

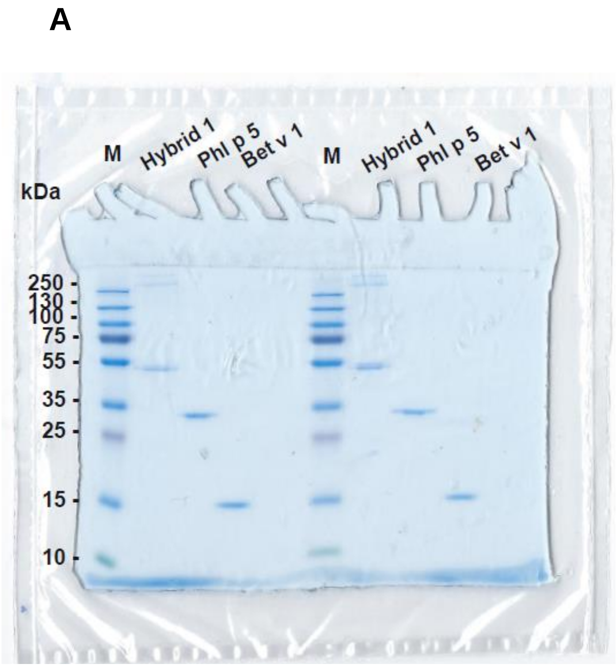

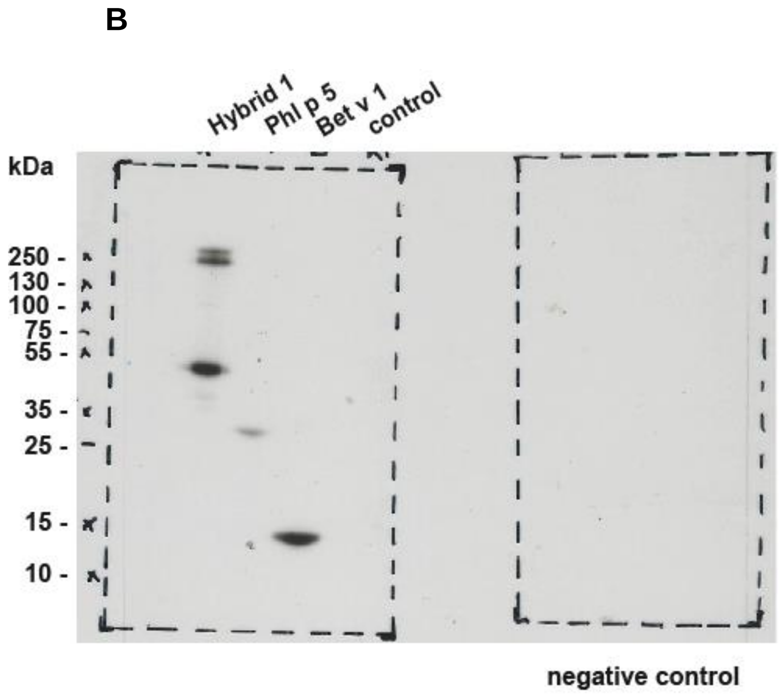

Supplementary figure S1

Supplementary Table 1

	cpm Bip 1	cpm 4A6	cpm Bip 1- cpm 4 A6	% difference in IgE
**Patient 1**	**626.3**	**367.3**	259.0	+71%
**Patient 2**	**544.6**	**613.3**	-68.7	-11%
**Patient 3**	**277.5**	**147.0**	130.5	+89%
**Patient 4**	**281.5**	**187.2**	94.3	+50%
**Patient 5**	**100.1**	**92.9**	7.2	+8%
**Patient 6**	**110.1**	**109.6**	0.5	+1%
**Patient 7**	**83.6**	**72.0**	11.6	+11%
**Patient 8**	**30.2**	**40.8**	-10.6	-26%
**Patient 9**	**75.4**	**71.3**	4.1	+6%
**Patient 10**	**42.9**	**59.2**	-16.3	-28%

Supplementary Table 2: Bet v 1-derived synthetic peptides

	position aa	sequence	Number of aa
Peptide 1	1-24	M G V F N Y E T E T T S V I P A A R L F K A F I **C**	25
Peptide 2	30-59	L F P K V A P Q A I S S V E N I E G N G G P G T I K K I S F **C**	31
Peptide 3	50-79	**C** G P G T I K K I S F P E G F P F K Y V K D R V D E V D H T N	31
Peptide 4	110-139	D G G S I L K I S N K Y H T K G D H E V K A E Q V K A S K E **C**	31
Peptide 5	130-160	**C** K A E Q V K A S K E M G E T L LR A V E S Y L L A H S D A Y N	32
Peptide 6	75-104	**C** V D H T N F K Y N Y S V I E G G P I G D T L E K I S N E I K	31

Position, sequence and length of Bet v 1-derived synthetic peptides
Boldface and underlined **C** indicates Cysteins added to facilitate coupling

Supplementary Table 3: Phl p 5-derived synthetic peptides

	position aa	sequence	Number of aa
Peptide 1	26-58	A D L G Y G P A T P A A P A A G Y T P A T P A A P A E A A P A G K **<u>C</u>**	34
Peptide 2	59-91	**<u>C</u>** A T T E E Q K L I E K I N A G F K A A L A A A A G V Q P A D K Y R	34
Peptide 3	93-128	**<u>C</u>** F V A T F G A A S N K A F A E G L S G E P K G A A E S S S K A A L T S K	37
Peptide 4	132-162	A Y K L A Y K T A EG A T P E A K Y D A Y V A T L S E A L R I **<u>C</u>**	32
Peptide 5	176-212	**<u>C</u>** A E E V K V I P A G E L Q V I E K V D A A F K V A A T A A N A A P A N D K	38
Peptide 6	217-246	**<u>C</u>** E A A F N D A I K A S T G G A Y E S Y K F I P A L E A A V K	31
Peptide 7	252-283	T V A T A P E V K Y T V F E T A L K K A I T A M S E A Q K A A K **<u>C</u>**	33

Position, sequence and length of Phl p 5-derived synthetic peptides
Boldface and underlined **<u>C</u>** indicates Cysteins added to facilitate coupling
